# Effect of exercise before and/or during taxane-containing chemotherapy treatment on chemotherapy-induced peripheral neuropathy symptoms in women with breast cancer: systematic review and meta-analysis

**DOI:** 10.1007/s11764-023-01450-w

**Published:** 2023-08-24

**Authors:** Rosiered Brownson-Smith, Samuel T. Orange, Nicola Cresti, Katherine Hunt, John Saxton, John Temesi

**Affiliations:** 1 Department of Sport, Exercise and Rehabilitation, Faculty of Health and Life Sciences, Northumbria University, Newcastle Upon Tyne, UK; 2 School of Biomedical, Nutritional and Sport Sciences, Faculty of Medical Sciences, Newcastle University, Newcastle uponTyne, UK; 3 Newcastle University Centre for Cancer, Newcastle University, Newcastle-Upon-Tyne, UK; 4 Northern Centre for Cancer Care, The Newcastle Upon Tyne Hospitals NHS Foundation Trust, Newcastle Upon Tyne, UK; 5 School of Sport, Exercise & Rehabilitation Sciences, University of Hull, Hull, UK

**Keywords:** Cancer, Neuropathy, Exercise, Chemotherapy, Quality of life, Fatigue

## Abstract

**Purpose:**

To systematically review and meta-analyse the efficacy of exercise interventions delivered before and/or during taxane-containing chemotherapy regimens on chemotherapy-induced peripheral neuropathy (CIPN), fatigue, and health-related quality of life (HR-QoL), in women with breast cancer.

**Methods:**

Seven electronic databases were systematically searched for randomised controlled trials (RCTs) reporting on the effects of exercise interventions in women with breast cancer receiving taxane-containing chemotherapeutic treatment. Meta-analyses evaluated the effects of exercise on CIPN symptoms, fatigue, and HR-QoL.

**Results:**

Ten trials involving exercise interventions ranging between 2 and 12 months were included. The combined results of four RCTs consisting of 171 participants showed a reduction in CIPN symptoms following exercise compared with usual care (standardised mean difference − 0.71, 95% CI − 1.24 to − 0.17, *p* = 0.012; moderate-quality evidence, *I*^2^ = 76.9%). Pooled results from six RCTs with 609 participants showed that exercise interventions before and/or during taxane-containing chemotherapy regimens improved HR-QoL (SMD 0.42, 95% CI 0.07 to 0.76, *p* = 0.03; moderate-quality evidence, *I*^2^ = 49.6%). There was no evidence of an effect of exercise on fatigue (− 0.39, 95% CI − 0.95 to 0.18, *p* = 0.15; very low-quality evidence, *I*^2^ = 90.1%).

**Conclusions:**

This systematic review found reduced levels of CIPN symptoms and an improvement in HR-QoL in women with breast cancer who exercised before and/or during taxane-based chemotherapy versus usual care controls.

**Implications for Cancer Survivors:**

This evidence supports the role of exercise as an adjunctive treatment for attenuating the adverse effects of taxane-containing chemotherapy on CIPN symptoms and HR-QoL.

**Supplementary Information:**

The online version contains supplementary material available at 10.1007/s11764-023-01450-w.

## Introduction

Due to advances in screening, early detection, and treatment, there are more people living with and beyond a breast cancer diagnosis than ever [1]. Major advancements in the treatment of cancer involve the use of modern chemotherapy, including cytotoxic agents such as platinum compounds and taxanes. However, their long- and short-term side effects can negatively impact a patients’ health-related quality of life (HR-QoL), during and after chemotherapy [2, 3]. This has prompted the need to investigate strategies to reduce the side-effects of treatment and improve HR-QoL.

Taxanes (e.g. paclitaxel, docetaxel) are among the most active cytotoxic chemotherapy drugs available for breast cancer [4]. The National Institute for Health and Clinical Excellence (NICE) recommends that taxanes are combined with anthracyclines for the treatment of invasive breast cancer, noting that the benefits of adding taxanes into a treatment regimen include reducing the risk of breast cancer recurrence and increasing survival rate [5]. However, taxanes also affect the structure and function of peripheral sensory, motor, and autonomic neurons [6], with the resultant impact often manifesting as chemotherapy-induced peripheral neuropathy (CIPN). Symptoms of CIPN include hand and foot numbness, paraesthesia, pain, and impairments to balance, gait, and posture [7]. Incidence rates of CIPN can range from 11 to 87%, depending on the specific drug and treatment regimen [8]. The burden of CIPN commonly results in dose-reduction and premature termination of treatment [9, 10]. These often severe symptoms can manifest alongside further debilitating side effects that can influence patient quality of life. Studies have found that 100% of patients receiving taxanes experienced fatigue [11, 12], with fatigue being the symptom experienced most severely [11]. Both cancer-related fatigue and CIPN symptoms are associated with reduced HR-QoL [13, 14].

Pharmacological interventions aimed at preventing CIPN have very limited evidence of efficacy [15, 16]. Duloxetine is the only currently recommended drug for paclitaxel-induced CIPN [17]; however, duloxetine use must be closely monitored by a physician and is associated with side effects (nausea, insomnia, and dizziness) [18]. Furthermore, a recent systematic review found duloxetine and placebo to be similar in efficacy [19]. Thus, an increasing body of research is being conducted into the impact of non-pharmacological interventions on CIPN and other common symptoms (including cancer-related fatigue) across a range of cancers. Exercise has been shown to enhance the expression of neurotrophic factors [20], reduce inflammation [21], and regulate mitochondrial dysfunction implicated in the development of CIPN [22–24]. A systematic review found that exercise during a variety of chemotherapy regimens improves CIPN symptoms and postural control [25], and exercise performed peri-chemotherapy, including after, reduces CIPN symptoms [26] and neuropathic pain [27]. Exercise during chemotherapy has also been shown to improve HR-QoL [25, 27], and exercise during adjuvant treatment for breast cancer reduces fatigue and cancer site-specific quality of life [28].

Although current data suggest that exercise has potential to alleviate symptoms of CIPN, available evidence syntheses have included exercise interventions prescribed to participants at any time point throughout their chemotherapy. Interventions that are given to participants post-chemotherapy could be tracking a natural easing of CIPN symptoms [29] and improvement of HR-QoL [30], therefore aiding rehabilitation as opposed to modulating CIPN symptom severity (and potentially, the underpinning neuropathology). Furthermore, previous reviews of exercise before and/or during chemotherapy have not investigated participants only receiving taxane-containing chemotherapy regimens. The mechanisms and symptoms of CIPN vary greatly across drug types, each potentially requiring unique management strategies [31, 32]. Moreover, CIPN can have important clinical implications for those receiving taxanes; 17% of those receiving taxanes require a dose reduction due to symptoms of CIPN specifically [10]. Taxane dose reduction is of significant concern as tumour control is associated with increased dose intensity [33]. Additionally, although CIPN severity often decreases gradually after treatment, symptoms frequently persist for at least 6 months after treatment cessation [14]. Therefore, identifying exercise as a potential adjunctive treatment to reduce the severity and/or risk of CIPN symptoms could benefit the immediate and long-term outcomes of treatment and reduce the impact of treatment well beyond chemotherapy termination. Thus, we systematically reviewed and meta-analysed the effect of exercise interventions before and/or during taxane-containing chemotherapy regimens on CIPN, fatigue, and HR-QoL in women undergoing breast cancer treatment.

## Methods

This systematic review was prospectively registered in the PROSPERO prospective register of systematic reviews (CRD42021272036) and followed the Preferred Reporting Items for Systematic Reviews and Meta-Analyses (PRISMA) guidelines [34]. There were some minor deviations from the study protocol, which are outlined and justified in Supplementary Material 1.

### Search strategy

An electronic search of PubMed, EMBASE, Cochrane Central, SPORTDiscus, CINAHL, ClinicalTrials.gov, and ISRTCN was run independently by two authors (RB-S, JT) from inception to 15th September 2022. Within the search, three key concepts were used, specifically breast cancer, exercise, and taxane-containing chemotherapy regimes in addition to their synonyms and controlled vocabulary (e.g. Medical Subject Headings). The search strategy used for each database is presented in Supplementary Material 2.

### Eligibility criteria

To be included in this review, studies had to be randomised control trials (RCTs) that recruited women with a breast cancer diagnosis, receiving any chemotherapy regimen containing taxanes. Participants had to have been ≥ 18 years old and randomised to either receive an exercise intervention before and/or during treatment or to usual care. We operationalised the control group as a group of participants that received standard care only or standard care plus the recommendation to follow general physical activity and/or healthy eating guidelines but did not receive the intended study intervention. Full-text articles in any language were eligible. It was required that outcomes included at least one of the following symptoms: CIPN, fatigue, or HR-QoL. Reviews, magazines, surveys, opinion pieces, commentaries, books, periodicals, editorials, conference abstracts, and case studies were excluded as were quasi-experimental, observational, and cross-over studies.

The exercise intervention must have been performed before and/or during the taxane-containing chemotherapy regimen. For the purpose of this review, exercise was defined as a subset of physical activity that is planned, structured, and repetitive and purposefully undertaken to improve health or fitness [35]. Interventions must have included a minimum of two exercise sessions and could have been aerobic, resistance, physical therapy, home-based, facility-based, unsupervised, or supervised. Exercise interventions could also have been given alongside a nutritional intervention. Included studies were required to provide data for a baseline assessment before any chemotherapy or exercise and a follow-up assessment immediately after chemotherapy termination. If the intervention continued beyond the end of chemotherapy, there must be data for included measures from an assessment point at the end of chemotherapy that could be compared to baseline.

### Outcomes

The outcomes included in this review were symptoms of CIPN, fatigue, and HR-QoL. The primary outcome was the difference in CIPN symptoms between intervention and usual care groups. For a CIPN outcome to be included in the systematic review, it must have been either generated from a CIPN-specific measure (e.g. EORTC QLQ-30 CIPN20) or be a previously reported, and specifically tested, CIPN symptom (e.g. balance). If not derived from a dedicated questionnaire, other measures of symptoms must have been explicitly assessing CIPN. Eligible symptoms included positive motor and sensory symptoms (hyperalgesia, allodynia, pain, dysesthesia, paraesthesia, muscle cramps, muscle aches) and negative motor and sensory symptoms (numbness, impaired fine motor skills, disturbance of vibratory and proprioceptive sensations, including balance and falls) [16, 36–40]. Secondary outcomes were differences in fatigue and HR-QoL. For a fatigue or HR-QoL outcome to be included in the systematic review, it must have been either a patient- or physician-reported index score, or subscale, of a fatigue or HR-QoL-specific assessment tool. The difference between baseline and follow-up scores from the intervention and usual care groups were compared for all outcomes.

### Study selection

After the completion of the literature searches, studies were collated into an Excel spreadsheet, and duplicates were removed by one reviewer (RB-S). Two reviewers (RB-S, JT) then independently screened titles and abstracts for eligibility. Full texts were then obtained for all studies that needed further assessment for eligibility. The same two reviewers then independently examined each full-text manuscript. Any disagreements were resolved via consensus meetings and consultation with a third author (STO).

### Data extraction

Data extraction was completed in duplicate by two reviewers (RB-S and JT) using a piloted data extraction form. The data items that were extracted from the included studies were authors, title, year of publication, study design, sample size, participant characteristics (e.g. age), treatment details, type and characteristics of the intervention and usual care groups, outcome measures, baseline and follow-up data (mean and SD), and rates of adherence to intervention. In the case of missing data, corresponding authors were contacted on at least two occasions within a 1-month period. If SDs were not reported, we collected other relevant data that could be converted to SDs, such as 95% confidence intervals (CIs) or *p*-values.

### Risk of bias

The Cochrane risk of bias tool for randomised trials (RoB2) [41] was used to assess the risk of bias for each study outcome of interest within each study. Judgements were made independently by two authors (RB-S, JT), with any disagreements being resolved by discussion and consensus. Availability of data was considered sufficient when there was data for 85% of randomised participants. This was based on the trial context potentially resulting in higher rates of dropout when compared to trials of pharmaceutical interventions.

When a meta-analysis included 10 or more effect sizes, the risk of bias due to missing results in a synthesis was explored with Egger’s test of the intercept [42] and by visually inspecting a funnel plot of the effect estimates plotted against their corresponding sampling variance.

### Quality of evidence

The quality of evidence found was assessed using the Grades of Recommendation, Assessment, Development, and Evaluation (GRADE) approach [43]. Risk of bias, inconsistency of results, indirectness of evidence, imprecision of results, and publication bias were assessed for each individual outcome. The evidence was downgraded by one level if judged to have a *serious limitation* or by two levels if judged to have a *very serious limitation*. GRADE assessments were performed by two independent authors (RB-S, JT), and conflicts were resolved through consensus.

### Statistical analysis

A meta-analysis of standardised mean differences (SMDs) between exercise and usual care groups was performed where two or more trials reported the same outcome. SMDs were calculated as the between-group difference in change scores (or difference in post-intervention scores if change scores were not available) divided by the pooled SD at baseline [44]. If SDs were not presented in the study, the SD was estimated from the reported standard error, 95% CI, or *p*-value. Qualitative descriptors used to interpret the strength of the SMDs were based on Cohen’s criteria [45] ( ±): trivial (< 0.2), small (0.2 to 0.49), moderate (0.5 to 0.79), and large (≥ 0.8).

Meta-analyses were performed with a random effect model using the inverse-variance method, where the weight of each study is the inverse of the variance of the effect estimate. The random effect model was chosen to incorporate potential heterogeneity. CIs and test statistics were calculated via a *t*-distribution using the Hartung-Knapp-Sidik-Jonkman (HKSJ) approach [46]. When a meta-analysis included more than one outcome measure from the same study, effect estimates were nested within studies using a multi-level structure to account for correlated effects [47].

A *χ*^2^ test was used to assess heterogeneity, with *p* < 0.1 indicating a significant degree of heterogeneity. The *I*^2^ statistic was then used to assess the percentage of variability in effect estimates due to heterogeneity rather than sampling error. The *I*^2^ thresholds used were in line with Cochrane guidelines; 0–40% (‘might not be important’), 30–60% (‘may represent moderate heterogeneity’), 50–90% (‘may represent substantial heterogeneity’), and 75–100% (‘considerable heterogeneity’) [48]. When a meta-analysis included 10 or more effect estimates and there was evidence of at least moderate heterogeneity, we performed meta-regressions to explore sources of heterogeneity, specifically the impact of the covariates (1) whether the outcome was objectively or subjectively measured and (2) whether the outcome assessed sensory symptoms or motor/autonomic symptoms.

We conducted sensitivity analyses on the main meta-analysis models to explore whether decisions made in the review process influenced the overall findings. Sensitivity analyses involved (1) computing test statistics and 95% CIs based on a normal (*z*) distribution rather than a *t*-distribution; (2) using imputed change-from-baseline SD to calculate effect estimates, rather than the SD at baseline; and (3) excluding studies where participants received chemoradiotherapy. Change-from-baseline SD was imputed using a correlation coefficient of 0.7 [49, 50]. We then performed a leave-one-out sensitivity analysis to assess whether removing an individual effect estimate from a meta-analysis influenced the model parameters.

Statistical analyses were conducted using R version 4.0.4 (R Foundation for Statistical Computing, Vienna, Austria). Statistical significance was set at *p* < 0.05. Data are presented as effect estimates with their corresponding 95% CI. The search results, dataset, and statistical analysis are available on Open Science Framework (OSF) repository (https://osf.io/bg896/?view_only=d70613ca97fb41689207a1a240b090df).

## Results

### Study selection

**Fig. 1:**
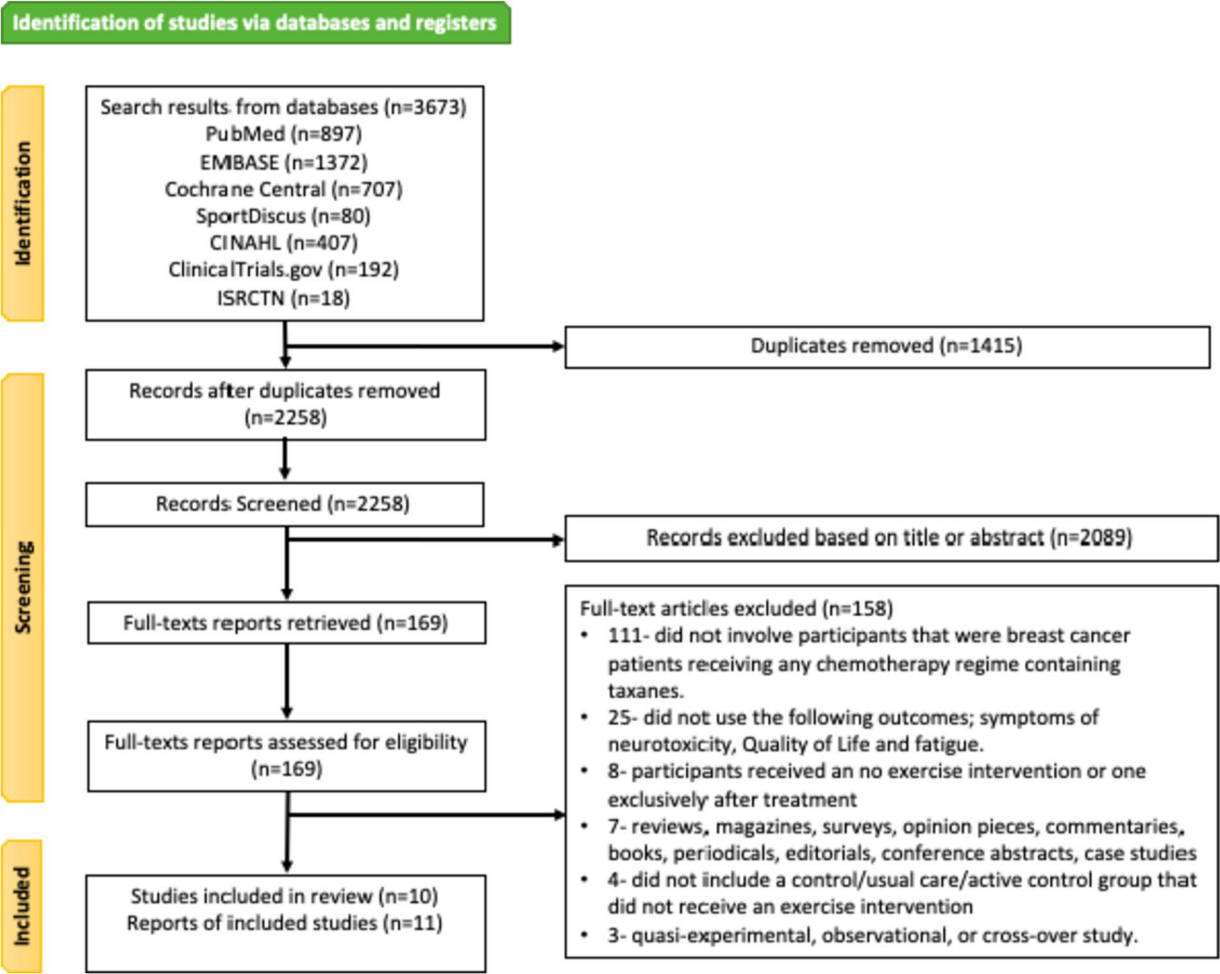
Summary of study selection process

The search of included databases generated 3673 results, of which 1415 were duplicates, and 2258 were screened by title and abstract. Full-text screening of 169 articles found 10 trials that met the eligibility criteria. One of the trials has data reported in two different papers [51, 52]. A summary of the study selection process is presented in Fig. 1.

### ‘Near misses’

A total of 32 studies were judged to meet many, but not all, of the eligibility criteria (i.e. ‘near misses’). The primary exclusion reason shared by all ‘near miss’ studies was the inclusion of participants receiving a mixture of chemotherapy types and the lack of a distinct dataset for those participants receiving taxane-containing chemotherapies. A full list of these studies and justifications for exclusion are presented in Supplementary Material 3.

### Study characteristics

A summary of general study characteristics is presented in Table 1. A total of 896 participants were included in this review, of which 171 were included in the primary CIPN meta-analysis, 737 included in the fatigue meta-analysis, and 609 included in the HR-QoL meta-analysis. Eight of the studies compared an exercise group to a usual care group [53–60], while two studies compared an immediate exercise group to a delayed exercise group that received usual care during the study [51, 52, 61]. One study included patients who had potentially received both chemotherapy and radiotherapy between baseline and post-intervention assessment [61]. Two of the studies were undertaken in Canada [51–53], one in Turkey [58], two in the USA [55, 59], one in Germany [60], and four in France [54, 56, 57, 61].

**Table 1:** Summary of study characteristics

Author(s) (year), country	Study design	Participants	Treatment details	Recruitment and assessment timing	*N*^a^, type of analysis	Age (years)
Andersen Hammond et al. [53] (2020), Canada	Two-arm RCT. 1: treatment (I), 2: control (C)	BC stage I-III. No comorbid conditions causing peripheral neuropathic symptoms	(1) Docetaxel 75 mg/m^2^ and cyclophosphamide 600 mg/m^2^ every 3 weeks for 4 cycles or (2) 5-fluorouracil 500 mg/m^2^, epirubicin 100 mg/m^2^, cyclophosphamide 500 mg/m^2^ every 3 weeks for 3 cycles, followed by docetaxel 100 mg/m^2^ given every 3 weeks for 3 cycles	Women were approached at their initial oncology visit. Data points included in meta-analysis: baseline, mid-CT, and post-CT	I: 22. C: 26. PP	I: x̄ 56.3 (± 9.9 SD). C: x̄ 53.0 (± 10.3 SD)
Bland et al. [51] (2019); Kirkham et al. [52] (2020), Canada	Two-arm RCT. 1 immediate exercise (I), 2: delayed exercise (C)	> 19 years. BC stage I-III. BMI < 40 kg/m^2^. No history of diabetes or neurologic disorders, acute or uncontrolled health conditions, or receipt of treatment for a past cancer diagnosis	Paclitaxel or docetaxel CT in 2- or 3-week cycles	The intervention began up to 1 week before the first taxane cycle and ended 2 or 3 weeks after the last cycle. Data points included in meta-analysis: baseline and end of CT	I^IE^: 12. C^DE^: 15. PP	I: x̄ 51.0 (± 8.1 SD). C: x̄ 49.5 (± 11 SD)
Carayol et al. [54] (2020), France	Two-arm RCT. 1: Adapted Physical Activity Diet (APAD)(I), 2: usual care (C)	18–75 years diagnosed with non-metastatic BC less than 6 months ago. No contraindications to moderate intensity physical activity, inability to attend intervention sessions or assessments, and a difficulty or disability preventing the patient from correctly understanding the trial information or requirement	6 cycles of adjuvant CT: FEC100 protocol for 3 cycles every 3 weeks, followed by docetaxel for 3 cycles every 3 weeks, followed by 6 weeks of radiotherapy	Enrolled after undergoing curative surgery and before CT. Data points included in meta-analysis: baseline and after CT (18 weeks)	I: 71. C: 64. PP	I: x̄ 52.1 (± 9.3 SD). C: x̄ 51.2 (± 10.9 SD)
Chaoul et al. [55] (2018), USA	Three-arm RCT. 1: Tibetan yoga programme (I), 2: stretching programme, 3: waitlist (C)	≥ 18 years. BC stage I-III. No lymphedema, deep vein thrombosis, thought disorder (e.g. schizophrenia), score of ≤ 23 on the mini-mental state examination, extreme mobility problems, or regular yoga practice	(1) Neoadjuvant or adjuvant paclitaxel given weekly for 12 cycles or every 3 weeks for 4 cycles, (2) neoadjuvant docetaxel given every 3 weeks for 4 cycles, followed by FAC/FEC every 3 weeks for 4 cycles	Women were approached either before starting or within the first 2 cycles of CT. Data points included in meta-analysis: baseline and 1 week after CT	I: 64. C: 79. PP	I: x̄ 49.5 (± 9.8 SD). C: x̄ 49.0 (± 10.1 SD)
Cornette et al. [56] (2016), France	Two-arm RCT. 1: Adapted Physical Activity (APA)(I), 2: usual care (C)	18–75 years treated with CT followed by radiotherapy	FEC100 protocol for 3 cycles every 3 weeks, followed by docetaxel for 3 cycles every 3 weeks	Data points included in meta-analysis: before CT (T0) and after CT (T1)	I: 22. C: 22 ITT with imputation	I: median 52 (37–73). C: median 49 (37–68)
Jacot et al. [57] (2020), France	Two-arm RCT. 1: Adapted Physical Activity Diet (APAD)(I), 2: control (C)	≥ 18 years. BC diagnosis < 6 months previously. No metastatic disease, other primary tumour or medical contraindications to moderate-intensity physical activity	FEC100 protocol followed by either docetaxel every 3 weeks or paclitaxel weekly for 9 weeks, followed by 6 weeks of radiotherapy. HER2-positive tumours also received adjuvant trastuzumab for a total of 52 weeks, starting at the initiation of taxane CT	Enrolled after undergoing curative surgery and before CT. Data points included in meta-analysis: baseline and after CT	I: 150. C: 157. ITT	I: x̄ 52.66 (± 9.69 SD). C: x̄ 52.35 (± 10.09 SD)
Simsek, Demir [58] (2021), Turkey	Three‐arm parallel RCT. 1: exercise (I), 2: cold application, 3: control (C)	≥ 18 years. > 1 neuropathy symptom according to CIPNAT. BC stage II-IV. No central nervous system issues (e.g. movement and balance, coordination, and sensation) or intolerance to cold	Weekly taxane group CT infusion dose of at least 70 mg/m^2^	Data points included in meta-analysis: pre and post CT	I: 30. C: 30. PP	I: 20–39 = 13.4%, 40–59 = 46.6%, 60 + = 40.0%. C: 20–39 = 26.7%, 40–59 = 53.3%, 60 + = 20.0%
Sturgeon et al. [59] (2022), USA	Two-arm RCT. 1: intervention (I), 2: control (C)	BC stage I-III. > 18 years. Sedentary defined as < 75 min/week of self-reported moderate intensity leisure-time physical activity over the past month. No presence of heart disease or previous history of anthracycline CT contraindications for exercise testing or training	(1) Taxotere, carboplatin, Herceptin + Perjeta [TCH + P], or (2) Adriamycin, cyclophosphamide, Taxol [ACT]	Data points included in meta-analysis: baseline and follow-up (after CT)	I: 8. C: 7. PP	I: x̄ 47.0 (± 11.7 SD). C: x̄ 51.5 (± 9.5 SD)
Vincent et al. [61] (2020), France	Three-arm RCT. 1: group A: 6-month home-based adapted physical activity (APA) programme during adjuvant or neoadjuvant therapy (I). 2: group B: 6-month home-based APA programme after adjuvant or neoadjuvant therapy (C). 3: group C: 12-month home-based APA programme during and after adjuvant or neoadjuvant therapy (I)	18–75 early stage BC treated with CT followed by radiotherapy. Normal initial left ventricular ejection fraction confirmed after CT if they were treated with trastuzumab. Women on hormone therapy who completed other primary cancer treatments were considered post-treatment. No symptomatic cardiac pulmonary disease or family history of sudden death in a first-degree relative or ongoing treatment with a beta-blocker	6 cycles of adjuvant or neoadjuvant CT; FEC100 protocol for 3 cycles every 3 weeks, followed by docetaxel for 3 cycles every 3 weeks, and trastuzumab for 12 months if the breast tumour was HER2 positive	A maximum of 15 days from baseline assessments to randomisation. Data points included in meta-analysis: before CT (T0) and after 6 months of treatment (T1)	I: group A (29) + group C (26) = 55. C: group B (26). ITT	I: group A—56.5 (minimum 30–maximum 69), group C—50.0 (minimum 29–maximum 72). C: group B—50.0 (min 37–max 72)
Vollmers et al. [60] (2018), Germany	Two-arm RCT. 1: intervention, 2: control	18–75 years. Primary BC. No existing cardiopulmonary disease, renal insufficiency, neurological disease, or metabolic disease	Primary paclitaxel treatment for 12 weeks	Data points included in meta-analysis: before paclitaxel and after last dose	I: 17. C: 19. PP	I: x̄ 48.56 (± 11.94 SD). C: x̄ 52.39 (± 10.14 SD)

*N*^a^ number of women included in the analysis of the primary outcome, *RCT* randomised controlled trial, *CT* chemotherapy, CIPNAT chemotherapy-induced peripheral neuropathy assessment tool, *BC* breast cancer, *FAC* 5-fluorouracil, doxorubicin, and cyclophosphamide, *FEC* epirubicin/cyclophosphamide/5-fluorouracil, *TC* docetaxel and cyclophosphamide, *I* intervention group, *C* control group, *IE* immediate exercise, *DE* delayed exercise, *PP* per-protocol, *ITT* intention-to-treat

There was a wide variety of exercise interventions included in this review. A summary of intervention characteristics is presented in Table 2. The duration of exercise interventions ranged from 2 to 12 months. One study did not report duration [55], one conducted the intervention for the duration of chemotherapy treatment and it continued for a further 6 weeks afterwards [60], and one commenced with chemotherapy and continued until symptoms of CIPN had subsided [53]. The type of exercise given to participants varied between studies, with some studies using a combination of resistance and aerobic exercises [51, 52, 54, 56, 57], combined strengthening, stretching, and balancing exercises [58], aerobic exercise via video recordings [59], or less strenuous physical training and sensorimotor exercises [60], Tibetan yoga [55], or nerve gliding exercises [53]. The frequency of interventions ranged from four sessions over the course of chemotherapy to three times daily, and session duration ranged from 5 to 90 min. Six studies provided some supervised sessions [51, 52, 54–58]. Nine of the studies had interventions during chemotherapy [53–61], and one began the intervention up to 1 week before chemotherapy [51, 52].

**Table 2:** Summary of interventions

Author(s) (year)	Type	Setting	Supervision	Duration of programme	Frequency (× /week)	Duration (min per session)	Intensity	Adherence (% unless stated otherwise)
Andersen Hammond et al. [53] (2020)	Nerve gliding exercises	H	UnS	To be completed until the symptoms of the neuropathy had subsided	3 × daily 7 × week	5–10	Not reported	Not reported
Bland et al. [51] (2019); Kirkham et al. [52] (2020)	AE and whole-body. RE 3/week, aerobic exercise 2/week	M	S 3/week, UnS 2/week	8–12 (matched to chemotherapy protocol)	5	S duration ranged from 25 to 40 min depending on time point in chemotherapy cycle. H-based exercise duration progressed from 15 to 30 min throughout	‘Chemotherapy-periodized’—intensity and duration dependent on time point in chemotherapy cycle. Week after chemotherapy—lower aerobic intensity (50–55% HRR) with increased duration (40 min). After first week of the chemotherapy cycle—intensity increased to 75% HRR by week 8. Duration progressed from 25 to 35 min on non-chemotherapy weeks. S AE modes included the treadmill, cycle ergometer, or elliptical trainer. RE: 5 specific exercises using machines—starting at 1 set of 10 repetitions at 50% of estimated 1 repetition maximum, progressing to 2 sets of 10 to 12 repetitions at 65% 1 repetition maximum. RE was reduced to 1 set per exercise for 1 week after chemotherapy	S exercise = 78 ± 23. H-based exercise = 87 ± 23
Carayol et al. [54] (2020)	RE (hamstrings, quadriceps, buttocks, abdominal, back, shoulders/arms) and AE (cycloergometer for S hospital-based, H-based performed via various modalities of aerobic exercise (e.g. walking) + 9 nutritional therapeutic education sessions	M, H, and hospital	Both S and UnS—3 × UnS weekly, 1 × S every 3 weeks	Approx. 26 weeks (data taken after 18 weeks)	3–1 RE, 2 AE + hospital-based S exercise sessions every 3 weeks (9 in total)	RE: 10-min warm-up, 2 to 5 (for each muscle group) sets with 6 to 12 repetitions. AE: 30–45 min	RE: 2 to 5 different tasks with increasing difficulty were available for each muscle group. Every 6 weeks, the exercise specialist proposed a 2-repetition or 1-set increase and/or shift for more difficult task. AE: 50–75% of the maximum heart rate	67
Chaoul et al. [55]	TYP: 4 main components: (1) mindfulness and focused attention, (2) an alternate nostril breathing practice and a breath retention exercise, (3) Tsa Lung movements, and 4) a brief compassion-based meditation	Yoga class	S-1–1 by TYP instructors that had at least 3 years of practice experience and received relevant oncology training	Not reported	4 times total during chemotherapy. Out of class practice was encouraged. Patients were provided materials and recordings of techniques	75 to 90	TYP movements are described as gentle	73
Cornette et al. [56] (2016)	AE (cycle ergometer or outside walking) and RE (resistance bands targeting abdominal, hamstring, quadriceps, triceps, and gluteus maximus) performed throughout adjuvant chemotherapy	H	UnS, however a specialist contacted patients by phone	27 weeks	Minimum 3	AE: 20 min initially, with an increase of 5 min every 6 weeks to achieve 40 min at the end of the programme. RE: two sets of 8–12 repetitions	AE: adapted to heart rate and power (cycling), as determined by the first VT of the CPET. Cycling speeds of 60 rpm were maintained	88
Jacot et al. [57] (2020)	One RE session and one AE each week. + 6 nutritional therapeutic education sessions	M, H, and hospital	8 S hospital-based exercise sessions and 44 UnS home-based sessions. One muscle strength session and one aerobic session each week	26 weeks. 15 of which are during chemotherapy	2	120 min per week	Each session consisted of 10 min of warm-up, at least 30 min of exercise, 10 min of stretching and 10 min of relaxation time. RE targeted six main muscle groups (hamstrings, quadriceps, buttocks, abdominal, back, shoulders/arms). Each skill was performed for 2 to 5 sets with 6 to 12 repetitions with individual adaptation and progression. S AE used a cycloergometer and H-based exercise consisted of various modalities for (e.g. walking)—exercise intensity began at 50–55% and progressed to 65–75% of the maximum heart rate by weeks 20–26	80
Simsek, Demir [58] (2021)	Strengthening and stretching exercises (foot dorsiflexion, foot plantar flexion, gastrocnemius stretching, hamstring stretching, quadriceps exercises, biceps, and hand flexion‐extension) followed by balance exercises (hip flexion, hip extension, hip abduction, and knee flexion)	H	S- by a researcher once a week, UnS 4/week	12 weeks	5	15–30	The exercise started with 10 repetitions for the first 3 weeks and increased to 20 repetitions in the second 3 weeks and increased to 30 repetitions in the last 3 weeks. Stretching was not repeated; 2 half‑litre water bottles were used during the exercise programme, and sheets and towels were used for support	Not reported
Sturgeon et al. [59] (2022)	AE—participants received commercially available aerobics DVDs and an informational binder of aerobic exercises. Participants were instructed to self-select the combination of activities that places them in their appropriate heart rate zone and were coached (via phone call) regarding this	H	UnS, however participants had phone calls with a coach 1 × /week that typically lasted 10–20 min	24 weeks	3	60 min/week–75 + min/week	Weeks 1–4—60 min/week at 50% of VO_2_max (RPE = 2) up to 75 + min/week at 60% of VO_2_max (RPE = 3–4). Weeks 5–11—increase exercise intensity from 60 to 80% VO_2_max. By week 11, the exercise prescription was 65–75% VO_2_max (RPE = 5–6) for 2 sessions per week and 80% + VO_2_max (RPE = 7–8) for 1 session. Weeks 12–24—exercise prescription from week 11 maintained	87.6
Vincent et al. [61] (2020)	AE (cycle ergometer) and RE (abdominal, hamstrings, quadriceps, triceps, and surae and gluteus maximus using elastic bands)	H	UnS, however a specialist contacted patients by phone weekly to check on progress and overcome any barriers to activity	A: 24 weeks, C: 48 weeks (12 weeks after, data taken at 24 weeks)	AE: > 2. RE: 1	AE: 57 min + (+ brisk walking. RE: The first session lasted 20 min and increased with increased repetitions	AE: 3 × 8 min at 60% of their max aerobic power, 1-min rest intervals + 30 min continuously at 70% + brisk walking if desired. RE: 2 sets of 8 initially, increased to 12 repetitions after an initial supervised session, 1 repetition was added every 6 weeks	AE ≥ 85%— A: 91%, B: 80%, C: 77%. RE training assessment performed—A: 66.8% of sessions (± 30.2). B: 84.2% (± 20.3), C: 74.4% (± 24.3)
Vollmers et al. [60] (2018)	Physical training (strength training of upper and lower extremity and a warmup endurance training) and sensorimotor exercises (based on balance training)	Not reported	UnS	18 weeks (12 during chemotherapy and 6 after)	2	Not reported	The strength training consisted of six different exercises which were executed twice with 20 repetitions. 13–15 on the Borg Scale. Dependent on patients overall physical status (age, weight, training state)	Not reported

*AE* aerobic exercise, *RE* resistance exercise, *TYP* Tibetan yoga programme, *H* home, *M* mixed setting, *S* supervised, *UnS* unsupervised, *HRR* heart rate reserve

### Quality of evidence

GRADE assessments showed that the quality of evidence for CIPN and HR-QoL was moderate. The meta-analysis data of both outcomes were judged to have, and downgraded for, serious imprecision due to low median sample size and a small number of included studies. The quality of evidence for fatigue was found to be very low. This was due to the high risk of bias found within one of the studies included in the fatigue meta-analysis, imprecision due to low sample size and a small number of included studies, and serious inconsistency of results. The results of the GRADE assessment can be found in Supplementary Material 4.

### Risk of bias

The risk of bias was evaluated for all outcomes included in the review (CIPN, fatigue, and HR-QoL). A common concern was the lack of blinding of outcome assessors, which resulted in consistent judgement of *some concerns* due to possible deviations from the intended interventions. An additional common source of bias was due to missing data from participant drop-out and a failure to correct for, or identify, any potential bias. Three studies conducted appropriate intention-to-treat analyses [56, 57, 61]; however, only one detailed a method of imputation [56]. The risk of bias for the fatigue meta-analysis was judged to be high due to high risk of bias in a single included study [55]. This judgement was the result of a high participant attrition rate. A summary of the results of the risk of bias assessment can be found in Supplementary Material 5.

### Effect on CIPN symptoms

**Fig. 2:**
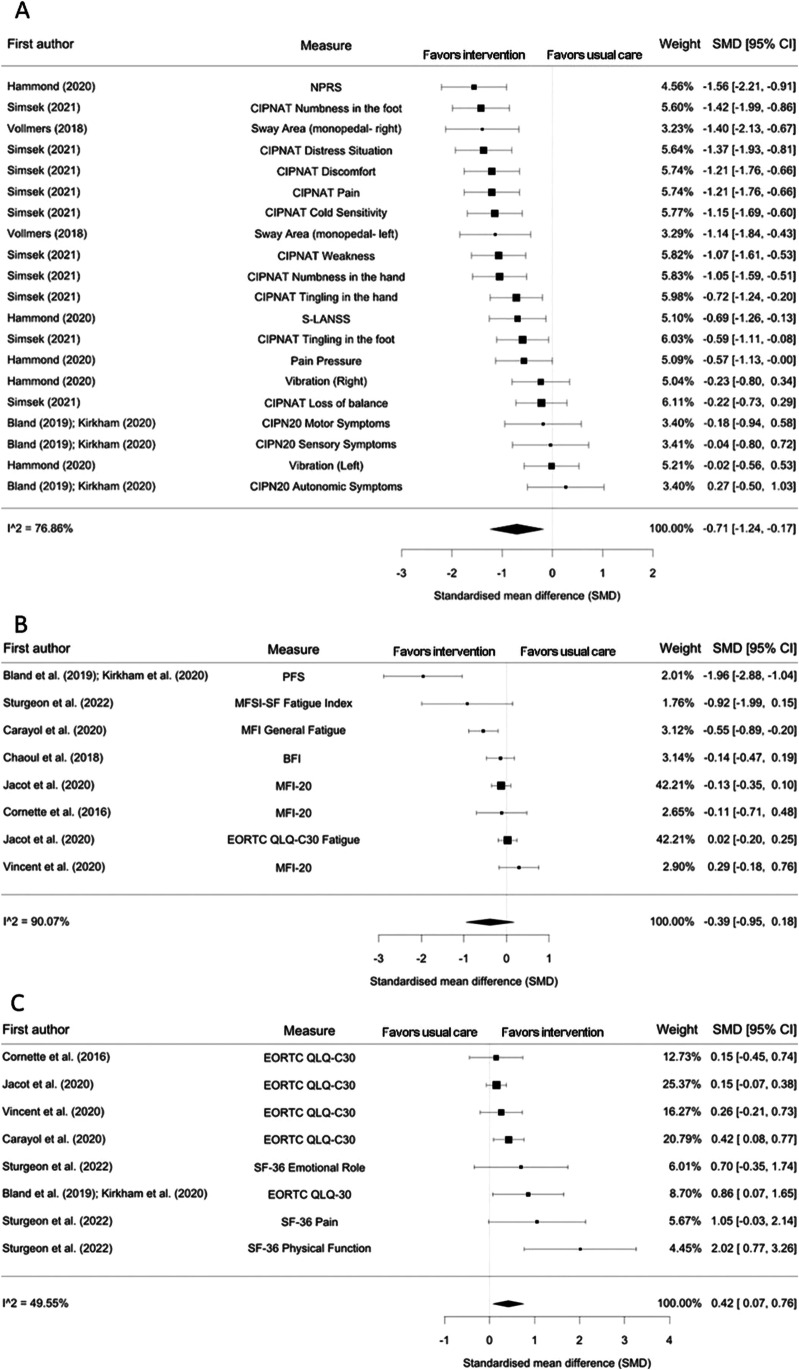
Forest plots of the results from multi-level random-effect meta-analyses on exercise intervention effects on **A** CIPN symptoms, **B** fatigue, and **C** HR-QoL. Data are presented as SMDs between exercise and usual care groups with corresponding 95% confidence intervals (95% CI). NPRS Numeric Pain Rating Scale, CIPNAT chemotherapy-induced peripheral neuropathy assessment tool, S-LANSS self-report version of leeds assessment for neuropathic symptoms and signs, CIPN20 The European Organisation for Research and Treatment of Cancer Quality of Life Questionnaire CIPN20, EORTC QLQ-30 The European Organisation for Research and Treatment of Cancer Quality of Life Questionnaire-30, SF-36 36-Item Short Form Health Survey questionnaire, PFS Piper Fatigue Scale, MFSI-SF Multidimensional Fatigue Symptom Inventory-Short Form, MFI Multidimensional Fatigue Inventory, BFI Brief Fatigue Inventory

The combined results of four RCTs [51–53, 58, 60] consisting of 20 effect estimates and 171 participants showed a reduction in CIPN symptoms following exercise compared with usual care (SMD − 0.71, 95% CI − 1.24 to − 0.17, *p* = 0.012; moderate-quality evidence; Fig. 2A). There was evidence of considerable heterogeneity (*I*^2^ = 76.9%, *p* < 0.001).

### Effect on fatigue

The pooled results from seven RCTs [51, 52, 54–57, 59, 61] consisting of 8 effect estimates and 737 participants showed no difference in the levels of fatigue between exercise and usual care groups (SMD − 0.39, 95% CI − 0.95 to 0.18, *p* = 0.15; very low-quality evidence; Fig. 2B). There was evidence of considerable heterogeneity (*I*^2^ = 90.1%, *p* < 0.001).

### Effect on HR-QoL

Based on the data from six RCTs [51, 52, 54, 56, 57, 59, 61] comprising 8 effect estimates and 609 participants, exercise interventions before and/or during taxane-containing chemotherapy regimens improved HR-QoL (SMD 0.42, 95% CI 0.07 to 0.76, *p* = 0.03; moderate-quality evidence; Fig. 2C). There was moderate heterogeneity of intervention effects (*I*^*2*^ = 49.6%, *p* = 0.06).

### Adverse events

Five studies found no serious adverse events related to the exercise intervention [51, 52, 54–56, 60]. One study reported no difference in exercise adverse events between control and intervention [59]. One study reported no grade 3 or 4 toxicity in patients in relation to the intervention, but 2 types of adverse events (fatigue and myalgia or arthralgia) for whom it was difficult to determine their origin (cancer, chemotherapy, or intervention). Tendinitis and a calf snap may have been associated with the intervention [61]. Three studies did not report adverse events related to the intervention [53, 57, 58].

### Sensitivity analysis

The use of a *z* distribution instead of a *t* distribution to compute the test statistics, change score SDs as the denominator in the calculation of SMDs instead of baseline SDs, and imputed change score SDs for the CIPN, fatigue, and HR-QoL meta-analyses, did not meaningfully influence the results. All sensitivity analyses can be found in Supplementary Material 6. The removal of each observation, in turn, had no significant impact on the effect estimate or level of heterogeneity in either the CIPN or fatigue meta-analysis. However, removing one effect estimate [54] changed the HR-QoL meta-analysis SMD so that it crossed the line of no effect. Removing Vincent et al. [61] from all meta-analyses, due to participants in that study receiving concomitant radiotherapy, did not impact the significance of any outcome. All results from the leave-one-out analysis are detailed in Supplementary Material 7.

### Meta-regressions

Meta-regressions are presented in Supplementary Material 8. The covariates had a negligible influence on the level of heterogeneity. Meta-regressions were not undertaken for fatigue or HR-QoL effects because the meta-analyses included less than 10 effect estimates.

## Discussion

This is the first study to synthesise data on the effects of exercise interventions before and/or during taxane-containing chemotherapy treatment on CIPN symptoms in women with breast cancer. This gives a unique insight into the potentially protective benefits of engaging in exercise during a taxane-containing chemotherapy regimen. Our findings show that performing exercise before and/or during taxane-containing regimens reduced symptoms of CIPN and improved HR-QoL. There was no evidence of an effect of exercise on fatigue. The evidence for CIPN and HR-QoL was judged to be of moderate quality, while the available evidence for the impact on fatigue was judged to be very low.

Several papers previously reported that exercise improves CIPN symptoms in patients with cancer [26]; however, previous evidence syntheses have not considered the potential therapeutic benefits of performing exercise before and/or during taxane-containing chemotherapy in women with breast cancer. Nevertheless, the finding that an exercise programme before and/or during taxane-containing chemotherapy regimens leads to higher levels of HR-QoL is consistent with the findings from a recent meta-analysis of RCTs that included a combination of treatment regimens, cancer types, and exercise intervention timings around chemotherapy (including after) [62].

The physiological mechanisms underpinning any preventive or attenuating effect of exercise on CIPN are currently unknown. However, the modelling of traumatic nerve injury in human and murine models has provided some mechanistic insight. For example, exercise has been shown to upregulate the expression of brain-derived neurotrophic factor (BDNF), insulin-like growth factor 1 (IGF-1) [20], and the anti-inflammatory cytokines IL-10 and IL-1RA [63, 64], potentially indicating a pathway of alleviating the nerve damage and/or attenuating inflammation that has been implicated in the aetiology of CIPN and its symptoms [65]. Furthermore, taxanes have been shown to induce opening of mitochondrial permeability transition pores (MPTP) in axons, leading to the loss of membrane potential, reduced ATP, mitochondrial swelling, increased reactive oxygen species, and calcium release [66]. Exercise can increase the antioxidant capacity and electron transport chain efficiency of mitochondria, and preclinical studies have shown that acute exercise increases the ability of mitochondria to accumulate Ca^2+^ before opening MPTP [22]. The understanding of psychosocial mechanisms underpinning any effect of exercise on CIPN is perhaps limited to the reported associations between exercise and mental health (e.g. improved mood, anxiety, depression) [65]. In this respect, exercise may exert some of its influence on CIPN by modulating the known relationship between CIPN, fatigue, anxiety, and depression [67, 68]. Furthermore, because pre-treatment anxiety is associated with higher incidence of CIPN development [68], reducing pre-treatment anxiety via exercise performed before and/or during treatment may at least partially explain any effect observed.

Although this systematic review gives a unique insight into the impact of exercise before and/or during chemotherapy, it has some important limitations. There were some minor deviations from the pre-registered protocol, all of which have been documented and justified in Supplementary Material 1. Additionally, a limited number of studies were eligible to be included in this review, which demonstrates a problematic lack of research into exercise before and/or during taxane-containing chemotherapy regimens on CIPN, fatigue, and HR-QoL. The risk of bias also highlighted *some concerns* in both the CIPN and HR-QoL meta-analyses, partly due to a lack of blinding. This factor is challenging to overcome due to the context of the research. However, although blinding is impossible, investigations into any potential bias that this may cause would be beneficial. The quality of evidence for the fatigue meta-analysis was judged to be very low due to a high risk of bias and serious inconsistency. Furthermore, it must be noted that the significance of the output of the HR-QoL meta-analysis was reliant on the inclusion of a single effect estimate [54], suggesting the need for further research to increase the robustness of this outcome. Therefore, additional high-quality evidence is required to fully evaluate the impact of an exercise intervention before and/or during taxane-containing chemotherapy on fatigue and HR-QoL levels.

Furthermore, the diversity of interventions and outcome measurements makes the convergence of data from the included studies challenging and led to considerable between-study heterogeneity. The number of sessions ranged from 4 in total throughout chemotherapy to 21 times a week (3 sessions daily) and session duration ranged from 15 to 90 min. This limits the relevance of the effect point estimates, as there may be considerable variation in the effectiveness of interventions. A number of the included studies used interventions that were a combination of muscle strengthening and aerobic exercise [51, 52, 54, 56, 57, 61] or muscle strengthening and stretching exercises [58] while others used exercise interventions having a much lower intensity such as nerve gliding exercises [53] and yoga [55]. High levels of clinical heterogeneity could provide a partial explanation for the statistical heterogeneity observed in all three meta-analyses. High clinical heterogeneity can also lead to inaccurate conclusions and ultimately mislead decision-making [69]. Finally, the diversity of outcome measurements could limit the power of combined results. The small number of eligible studies rendered pooling only those studies that had the same outcome measures impossible. However, we chose a priori to incorporate potential heterogeneity into a random effects model under the assumption that the effects of exercise on different CIPN symptoms would be different, yet related, and would follow a normal distribution. The issue of varied and subjective CIPN measurements is one that appears in clinical practice. When interviewed, clinicians have previously expressed that one of the main barriers to CIPN assessment and management was ‘CIPN assessment practice patterns (e.g. use of subjective instead of objective CIPN assessment approaches)’ [70]. Therefore, increasing consistency in CIPN measurement will benefit research convergence as well as active clinical management. Future studies should focus on maximising evidence quality, by limiting the impact of missing data, working to reduce bias due to lack of participant blinding by blinding outcome assessors and data analysts, increasing the use of patient-centred measures, and striving towards a consistent and holistic CIPN measure.

A key strength of this evidence synthesis was the rigorous methodological approach, which included multiple sensitivity analyses of the main meta-analysis findings and a leave-one-out analysis, to individually assess the impact of each included observation. Additionally, heterogeneity was explored using meta-regressions were appropriate. The protocol and analysis plan were prospectively registered in the PROSPERO prospective register of systematic reviews (ref: CRD42021272036), and the search results, data, and statistical code are publicly available on OSF. Furthermore, we did not restrict the literature search to manuscripts only available in English, thus reducing the chance of missing any relevant studies written in other languages.

## Conclusion

This review found reduced levels of CIPN symptoms and a higher HR-QoL in women with breast cancer who exercised before and/or during taxane-containing chemotherapy regimens, when compared to a usual care group. In contrast, there was no evidence of an effect of exercise on fatigue. Therefore, these results support the use of exercise, as an adjunct treatment before and/or during a taxane-containing treatment regimen for breast cancer, to reduce CIPN symptoms and improve HR-QoL.

## Supplementary Information

Below is the link to the electronic supplementary material.

Supplementary file1 (DOCX 474 KB)

## Data Availability

All data analysed during the meta-analyses and code used are available on the Open Science Framework (https://osf.io/bg896/?view_only=d70613ca97fb41689207a1a240b090df).
