# Supplementary material for: Effect of exercise before and/or during taxane-containing chemotherapy treatment on chemotherapy-induced peripheral neuropathy symptoms in women with breast cancer: systematic review and meta-analysis
Source: J Cancer Surviv. 2023 Aug 24;19(1):78–96. doi: 10.1007/s11764-023-01450-w (PMC11813970; doi:10.1007/s11764-023-01450-w)
Supplement: Supplementary file 1 — Supplementary file1 (DOCX 474 KB) [file 11764_2023_1450_MOESM1_ESM.docx]

**Supplementary Material 1: Deviations from the study pre-registered protocol (PROSPERO)**

| **Protocol method** | **Deviation from protocol method, with justfication** |
| --- | --- |
| We planned to use the following search syntax to identify studies with exercise interventions: *“Exercise (Mesh) OR exercise*(tlab) OR “physical activit*”(tlab) OR*  *training(tlab) OR sport*(tlab) OR aerobic*(tlab) OR “strength train*”(tlab) OR*  *walking*(tlab) OR swim*(tlab) OR cycl*(tlab) OR kinesiotherapy(tlab) OR*  *kinesitherapy(tlab) OR “resistance training”(tlab) OR weightlift*(tlab)”* | We changed the search syntax for exercise to *“Exercise (Mesh) OR exercise*(tlab) OR “physical activit*”(tlab) OR sport*(tlab)*  *OR aerobic*(tlab) OR “strength train*”(tlab) OR walking*(tlab) OR “resistance*  *training”(tlab) OR weightlift*(tlab)”.* This was done because to reduce the large amount of irrelevant search results. |
| We did not plan to do sensitivity analyses. | We undertook the following post-hoc sensitivity analyses:  (1) test statistics and 95% CIs based on a normal (z) distribution rather than a t-distribution, (2) imputed change-from-baseline SD to calculate effect estimates, rather than the SD at baseline, (3) exclusion of studies where participants received chemoradiotherapy, and (4) Leave-One-Out analysis to explore the influence of decisions made in the planning process and impact of each individual observation. |
| We did not plan to do meta-regressions. | When a meta-analysis included 10 or more effect estimates and there was evidence of at least moderate heterogeneity, we performed a meta-regression to explore sources of heterogeneity. Covariates included: 1) whether the measure was objective or subjective, 2) whether the measure was the measure was sensory or other (e.g., motor).  We did this due to explore the large amount of heterogeneity that was observed. |

**Supplementary Material 2: Search strategy**

|  |  |
| --- | --- |
|  | **Cochrane** |
| 1 | “breast neopla*” OR “breast cancer*” OR “breast tumo*” OR “breast carcinoma*” OR “breast adenocarcinoma*” OR “breast sarcoma*”. |
| 2 | exercise* OR “physical activit*” OR sport* OR aerobic* OR “strength train*” OR “resistance training” OR weightlift* |
| 3 | chemotherap* OR anti-neopla* OR abraxane OR taxane* OR docetaxel OR paclitaxel OR taxol OR taxotere OR Onxol |
| 4 | 1 AND 2 AND 3 Tl/AB/KW |
|  | |
|  | **PubMed** |
| 1 | Breast neoplasm (MeSH) OR “breast neopla*” OR “breast cancer*” OR “breast tumo*” OR “breast carcinoma*” OR “breast adenocarcinoma*” OR “breast sarcoma*” |
| 2 | Exercise (Mesh) OR exercise* OR “physical activit*” OR sport* OR aerobic* OR “strength train*” OR walking* OR “resistance training” OR weightlift* |
| 3 | \| Drug Therapy (Mesh) OR chemotherap* OR anti-neopla* OR abraxane OR taxane* OR docetaxel OR paclitaxel OR taxol OR taxotere OR Onxol \| \| --- \| |
| 4 | 1 AND 2 AND 3  All non-MeSH terms TL/AB  Filter: Human |
|  | |
|  | **CINAHL** |
| 1 | “breast neopla*” OR “breast cancer*” OR “breast tumo*” OR “breast carcinoma*” OR “breast adenocarcinoma*” OR “breast sarcoma*” |
| 2 | exercise* OR “physical activit*” OR sport* OR aerobic* OR “strength train*” OR “resistance training” OR weightlift* |
| 3 | chemotherap* OR anti-neopla* OR abraxane OR taxane* OR docetaxel OR paclitaxel OR taxol OR taxotere OR Onxol |
|  | 1 AND 2 AND 3 |
|  | |
|  | **ClinicalTrials.Gov** |
| Condition | Breast Cancer |
| Other Terms | exercise OR physical activity OR sport OR aerobic OR strength training OR resistance training OR weightlifting |
| Study Type | Completed Studies \| Interventional Studies |
|  | |
|  | **EMBASE** |
| 1 | “breast neopla*” OR “breast cancer*” OR “breast tumo*” OR “breast carcinoma*” OR “breast adenocarcinoma*” OR “breast sarcoma*” |
| 2 | exercise* OR “physical activit*” OR sport* OR aerobic* OR “strength train*” OR “resistance training” OR weightlift* |
| 3 | \| chemotherap* OR anti-neopla* OR abraxane OR taxane* OR docetaxel OR paclitaxel OR taxol OR taxotere OR Onxol \| \| --- \| |
|  | 1 AND 2 AND 3 in abstract |
|  | |
|  | **SPORTDiscus** |
| 1 | “breast neopla*” OR “breast cancer*” OR “breast tumo*” OR “breast carcinoma*” OR “breast adenocarcinoma*” OR “breast sarcoma*” |
| 2 | exercise* OR “physical activit*” OR sport* OR aerobic* OR “strength train*” OR “resistance training” OR weightlift* |
| 3 | \| chemotherap* OR anti-neopla* OR abraxane OR taxane* OR docetaxel OR paclitaxel OR taxol OR taxotere OR Onxol \| \| --- \| |
|  | 1 AND 2 AND 3 |
|  | |
|  | **ISRCTN** |
|  | Condition: Breast cancer, Interventions: Exercise, Trial Status: Ongoing |

**Supplementary Material 3: “Near miss” studies with reasons**

| Reference | Title | Reason |
| --- | --- | --- |
| Al-Majid et al. [1] | Effects of exercise on biobehavioral outcomes of fatigue during cancer treatment: results of a feasibility study | No distinct taxane group indicated |
| Ariza-Garcia et al. [2] | A Web-Based Exercise System (e-CuidateChemo) to Counter the Side Effects of Chemotherapy in Patients with Breast Cancer: Randomized Controlled Trial | No distinct taxane group indicated |
| Basen-Engquist et al. [3] | Feasibility and efficacy of a weight gain prevention intervention for breast cancer patients receiving neoadjuvant chemotherapy: a randomized controlled pilot study | No distinct taxane group indicated |
| Bolam et al. [4] | Two-year follow-up of the OptiTrain randomised controlled exercise trial | No distinct taxane group indicated |
| Campbell et al. [5] | A pilot study of a supervised group exercise programme as a rehabilitation treatment for women with breast cancer receiving adjuvant treatment | No distinct taxane group indicated |
| Cešeiko et al. [6] | Heavy Resistance Training in Breast Cancer Patients Undergoing Adjuvant Therapy | No distinct taxane group indicated |
| Courneya et al. [7] | Six-month follow-up of patient-rated outcomes in a randomized controlled trial of exercise training during breast cancer chemotherapy | No distinct taxane group indicated |
| Demark-Wahnefried et al. [8] | Results of a diet/exercise feasibility trial to prevent adverse body composition change in breast cancer patients on adjuvant chemotherapy | No distinct taxane group indicated |
| Haines et al. [9] | Multimodal exercise improves quality of life of women being treated for breast cancer, but at what cost? Randomized trial with economic evaluation | No distinct taxane group indicated |
| Hammer et al. [10] | Prescribed Walking for Glycemic Control and Symptom Management in Patients Without Diabetes Undergoing Chemotherapy | No distinct taxane group indicated |
| He et al. [11] | Rehabilitation Effect of Systematic Exercise in Adjuvant Chemotherapy for Breast Cancer | No distinct taxane group indicated |
| He et al. [12] | Effects of a 16-week dance intervention on the symptom cluster of fatigue-sleep disturbance-depression and quality of life among patients with breast cancer undergoing adjuvant chemotherapy: A randomized controlled trial | No distinct taxane group indicated |
| Hiensch et al. [13] | Inflammation Mediates Exercise Effects on Fatigue in Patients with Breast Cancer | No distinct taxane group indicated |
| Huang et al. [14] | The effect of a 12-week home-based walking program on reducing fatigue in women with breast cancer undergoing chemotherapy: A randomized controlled study | No distinct taxane group indicated |
| Husebø et al. [15] | Effects of scheduled exercise on cancer-related fatigue in women with early breast cancer | No distinct taxane group indicated |
| Mijwel et al. [16] | High-intensity exercise during chemotherapy induces beneficial effects 12 months into breast cancer survivorship | No distinct taxane group indicated |
| Mock et al. [17] | Exercise manages fatigue during breast cancer treatment: a randomized controlled trial | No distinct taxane group indicated |
| Mock et al. [18] | Fatigue and quality of life outcomes of exercise during cancer treatment | No distinct taxane group indicated |
| Moros et al. [19] | [Effects of an exercise training program on the quality of life of women with breast cancer on chemotherapy] | No distinct taxane group indicated |
| Mutrie et al. [20] | Five-year follow-up of participants in a randomised controlled trial showing benefits from exercise for breast cancer survivors during adjuvant treatment. Are there lasting effects? | No distinct taxane group indicated |
| Naraphong et al. [21] | Exercise intervention for fatigue-related symptoms in Thai women with breast cancer: A pilot study | No distinct taxane group indicated |
| Prakash et al. [22] | Effectiveness of yoga on quality of life of breast cancer patients undergoing chemotherapy: a randomized clinical controlled study | No distinct taxane group indicated |
| Schmidt et al. [23] | Effects of resistance exercise on fatigue and quality of life in breast cancer patients undergoing adjuvant chemotherapy: A randomized controlled trial | No distinct taxane group indicated |
| Schmidt et al. [24] | Comparing Endurance and Resistance Training with Standard Care during Chemotherapy for Patients with Primary Breast Cancer | No distinct taxane group indicated |
| Segal et al. [25] | Structured exercise improves physical functioning in women with stages I and II breast cancer: results of a randomized controlled trial | No distinct taxane group indicated |
| Smith-Turchyn et al. [26] | Bridging the gap: incorporating exercise evidence into clinical practice in breast cancer care | No distinct taxane group indicated |
| Taso et al. [27] | The effect of yoga exercise on improving depression, anxiety, and fatigue in women with breast cancer: a randomized controlled trial | No distinct taxane group indicated |
| van Waart et al. [28] | Effect of Low-Intensity Physical Activity and Moderate- to High-Intensity Physical Exercise During Adjuvant Chemotherapy on Physical Fitness, Fatigue, and Chemotherapy Completion Rates: Results of the PACES Randomized Clinical Trial | No distinct taxane group indicated |
| Wang et al. [29] | [Effect of Yoga on cancer related fatigue in breast cancer patients with chemotherapy] | No distinct taxane group indicated |
| Wang [30] | Effects of a six-week home-based walking program on Taiwanese women newly diagnosed with early stage breast cancer | No distinct taxane group indicated |
| Wei et al. [31] | Effects of Baduanjin exercise on cognitive function and cancer-related symptoms in women with breast cancer receiving chemotherapy: a randomized controlled trial | No distinct taxane group indicated |
| Yang et al. [32] | Effects of a home-based walking program on perceived symptom and mood status in postoperative breast cancer women receiving adjuvant chemotherapy | No distinct taxane group indicated |

**Supplementary Material 4: GRADE assessment**

| **Summary of findings** | | | | | **Quality assessment** | | | |
| --- | --- | --- | --- | --- | --- | --- | --- | --- |
| **Outcome** | **No. of participants (studies)** | **Pooled SMD (95% CI)** | **Risk of Bias** | **Inconsistency** | **Indirectness** | **Imprecision** | **Publication bias** | **Quality rating** |
| **CIPN** | 171 (4) | -0.71  (-1.24, -0.17) | Some concerns | No serious inconsistency | No serious indirectness | Serious imprecision | Undetected | Moderate |
| **Quality of Life** | 609 (6) | 0.42  (0.07, 0.76) | Some concerns | No serious inconsistency | No serious indirectness | Serious imprecision | Undetected | Moderate |
| **Fatigue** | 737 (7) | -0.38  (-0.95, 0.18) | High | Serious inconsistency | No serious indirectness | Serious imprecision | Undetected | Very Low |

**Supplementary material 5: ROB2 assessments**

*
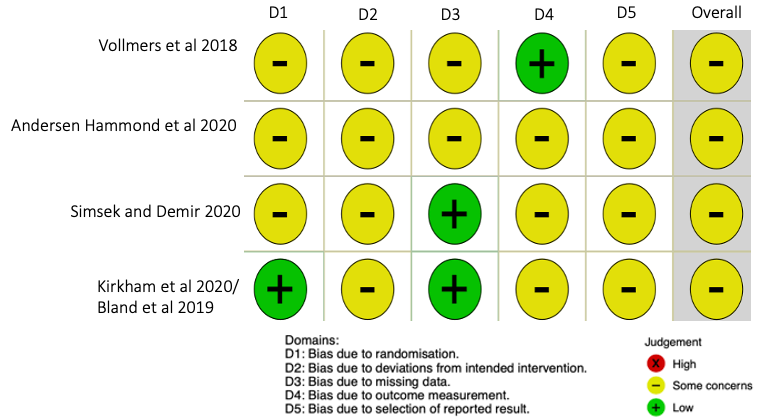
CIPN*

**
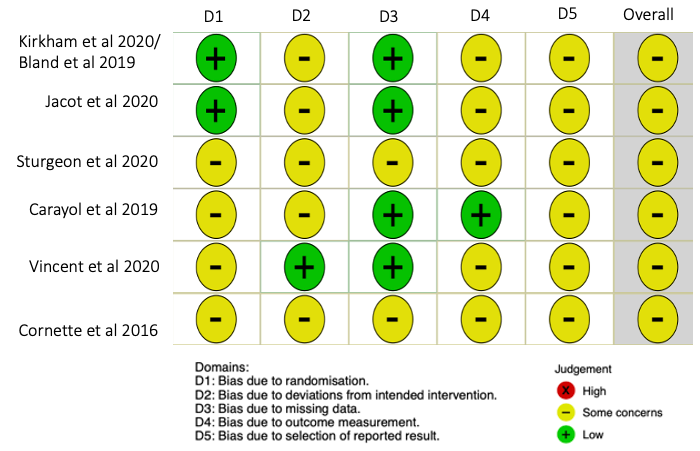
***QoL*

*Fatigue*

*
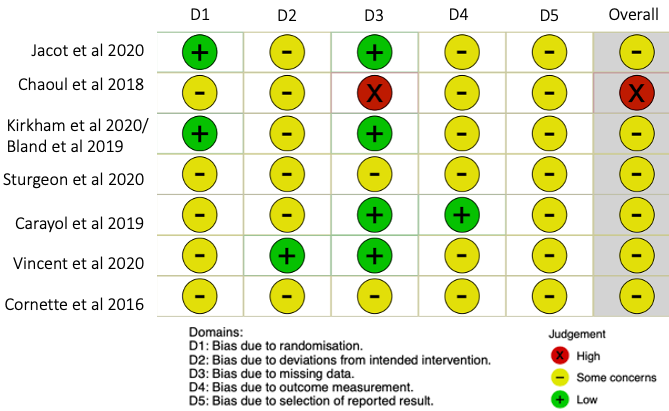
*

**Supplementary Material 6: Sensitivity analyses**

| **Sensitivity analysis** | **SMD (95% CI)** | *I^2^* |
| --- | --- | --- |
| **CIPN** |  |  |
| Z distribution | -0.71 (-1.21 to -0.21) | 76.9% |
| Change Score SD | -0.82 (-1.46 to -0.18) | 84.5% |
| **QoL** |  |  |
| Z distribution | 0.42 (0.13 to 0.70) | 49.6% |
| Change Score SD | 0.54 (0.06 to 1.03) | 73.1% |
| **Fatigue** |  |  |
| Z distribution | -0.39 (-0.86 to 0.08) | 90.1% |
| Change Score SD | -0.49 (-1.24 to 0.26) | 94.6% |

**Supplementary Material 7: Leave-one-out analyses**

| Leave-one-out sensitivity analyses on each meta-analysed outcome | | | |
| --- | --- | --- | --- |
| **Study omitted** | **Effect omitted** | **SMD (95% CI)** | *I^2^* |
| **CIPN** |  |  |  |
| None | - | -0.71 (1.24 to -0.17) | 76.9% |
| Simsek, Demir (2021) | Numbness in the hand | -0.71 (-1.24 to -0.17) | 77.0% |
| Simsek, Demir (2021) | Numbness in the foot | -0.70 (-1.21 to -0.17) | 75.9% |
| Simsek, Demir (2021) | Tingling in the hand | -0.72 (-1.26 to -0.17) | 77.4% |
| Simsek, Demir (2021) | Tingling in the foot | -0.72 (-1.27 to -0.17) | 77.4% |
| Simsek, Demir (2021) | Discomfort | -0.70 (-1.23 to -0.17) | 76.6% |
| Simsek, Demir (2021) | Cold Sensitivity | -0.70 (-1.23 to -0.17) | 76.8% |
| Simsek, Demir (2021) | Pain | -0.70 (-1.23 to -0.17) | 76.6% |
| Simsek, Demir (2021) | Weakness | -0.70 (-1.23 to -0.17) | 77.0% |
| Simsek, Demir (2021) | Loss of balance | -0.73 (-1.23 to -0.16) | 76.6% |
| Simsek, Demir (2021) | Distress Situation | -0.70 (-1.22 to -0.17) | 76.1% |
| Vollmers et al. (2018) | Sway Area (monopedal- right) | -0.66 (-1.16 to -0.17) | 74.9% |
| Vollmers et al. (2018) | Sway Area (monopedal- left) | -0.70 (-1.25 to -0.14) | 77.7% |
| Bland et al. (2019); Kirkham et al. (2020) | CIPN20 Sensory Symptoms | -0.72 (-1.24 to -0.22) | 75.8% |
| Bland et al. (2019); Kirkham et al. (2020) | CIPN20 Motor Symptoms | -0.71 (-1.26 to -0.16) | 78.0% |
| Bland et al. (2019); Kirkham et al. (2020) | CIPN20 Autonomic Symptoms | -0.76 (-1.20 to -0.33) | 70.9% |
| Andersen Hammond et al. (2020) | S-LANSS | -0.70 (-1.24 to -0.16) | 77.5% |
| Andersen Hammond et al. (2020) | Vibration (Left) | -0.75 (-1.28 to -0.22) | 75.7% |
| Andersen Hammond et al. (2020) | Vibration (Right) | -0.73 (-1.26 to -0.20) | 76.6% |
| Andersen Hammond et al. (2020) | Pain Pressure | -0.71 (-1.25 to -0.17) | 77.3% |
| Andersen Hammond et al. (2020) | NPRS | -0.65 (-1.23 to -0.07) | 77.5% |
|  | | | |
| **QoL** |  |  |  |
| None |  | 0.42 (0.07 to 0.76) | 49.6% |
| Bland et al. (2019); Kirkham et al. (2020) | EORTC QLQ-30 | 0.37 (0.01 to 0.73) | 48.4% |
| Jacot et al. (2020) | EORTC QLQ-30 | 0.51 (0.09 to 0.93) | 38.4% |
| Sturgeon et al. (2022) | SF-36 Pain | 0.37 (0.02 to 0.67) | 36.4% |
| Sturgeon et al. (2022) | SF-36 Emotional Role | 0.43 (0.05 to 0.80) | 56.3% |
| Sturgeon et al. (2022) | SF-36 Physical Function | 0.32 (0.07 to 0.56) | 16.9% |
| Carayol et al. (2020) | EORTC QLQ-30 | 0.45 (-0.03 to 0.93) | 56.7% |
| Vincent et al. (2020) | EORTC QLQ-30 | 0.47 (0.02 to 0.93) | 59.8% |
| Cornette et al. (2016) | EORTC QLQ-30 | 0.48 (0.03 to 0.91) | 58.8% |
|  | | | |
| **Fatigue** |  |  |  |
| None |  | -0.38 (-0.95 to 0.18) | 90.1% |
| Chaoul et al. (2018) | BFI | -0.46 (-1.19 to 0.27) | 92.1% |
| Bland et al. (2019); Kirkham et al. (2020) | PFS | -0.16 (-0.47 to 0.15) | 61.9% |
| Jacot et al. (2020) | MFI-20 | -0.38 (-0.98 to 0.22) | 88.1% |
| Jacot et al. (2020) | EORTC QLQ-C30 Fatigue | -0.40 (-0.98 to 0.18) | 87.0% |
| Sturgeon et al. (2022) | MFSI-SF Fatigue Index | -0.34 (-0.97 to 0.30) | 91.8% |
| Carayol et al. (2020) | MFI General Fatigue | -0.38 (-1.11 to 0.35) | 92.3% |
| Vincent et al. (2020) | MFI-20 | -0.50 (-1.13 to 0.11) | 90.0% |
| Cornette et al. (2016) | MFI-20 | -0.45 (-1.17 to 0.26) | 92.9% |

**Supplementary Material 8:** CIPN Meta-regression details and output

| **Objective** | **Sensory Symptom** |
| --- | --- |
| 1= objective  0= subjective | 1= Sensory  0= other |

| **Covariate** | **Coefficient (95% CI)** | ***p*-value** | ***I^2^* (χ2 *p*-value)** |
| --- | --- | --- | --- |
| **Objective**  Yes  No | 0.63 (-0.06 to 1.32) | 0.07 | 86.62% (< .01) |
| **Sensory Symptom**  Yes  No | -0.26 (-0.82 to 0.29) | 0.33 | 78.43% (< .01) |

**References**

1. Al-Majid S, Wilson LD, Rakovski C, Coburn JW. Effects of exercise on biobehavioral outcomes of fatigue during cancer treatment: results of a feasibility study. Biol Res Nurs. 2015;17(1):40-8. doi: 10.1177/1099800414523489.

2. Ariza-Garcia A, Lozano-Lozano M, Galiano-Castillo N, Postigo-Martin P, Arroyo-Morales M, Cantarero-Villanueva I. A Web-Based Exercise System (e-CuidateChemo) to Counter the Side Effects of Chemotherapy in Patients With Breast Cancer: Randomized Controlled Trial. J Med Internet Res. 2019;21(7):e14418. doi: 10.2196/14418.

3. Basen-Engquist KM, Raber M, Carmack CL, Arun B, Brewster AM, Fingeret M, et al. Feasibility and efficacy of a weight gain prevention intervention for breast cancer patients receiving neoadjuvant chemotherapy: a randomized controlled pilot study. Support Care Cancer. 2020;28(12):5821-32. doi: 10.1007/s00520-020-05411-2.

4. Bolam KA, Mijwel S, Rundqvist H, Wengström Y. Two-year follow-up of the OptiTrain randomised controlled exercise trial. Breast Cancer Res Treat. 2019;175(3):637-48. doi: 10.1007/s10549-019-05204-0.

5. Campbell A, Mutrie N, White F, McGuire F, Kearney N. A pilot study of a supervised group exercise programme as a rehabilitation treatment for women with breast cancer receiving adjuvant treatment. Eur J Oncol Nurs. 2005;9(1):56-63. doi: 10.1016/j.ejon.2004.03.007.

6. Cešeiko R, Thomsen SN, Tomsone S, Eglītis J, Vētra A, Srebnijs A, et al. Heavy Resistance Training in Breast Cancer Patients Undergoing Adjuvant Therapy. Med Sci Sports Exerc. 2020;52(6):1239-47. doi: 10.1249/mss.0000000000002260.

7. Courneya KS, Segal RJ, Gelmon K, Reid RD, Mackey JR, Friedenreich CM, et al. Six-month follow-up of patient-rated outcomes in a randomized controlled trial of exercise training during breast cancer chemotherapy. Cancer Epidemiol Biomarkers Prev. 2007;16(12):2572-8. doi: 10.1158/1055-9965.Epi-07-0413.

8. Demark-Wahnefried W, Case LD, Blackwell K, Marcom PK, Kraus W, Aziz N, et al. Results of a diet/exercise feasibility trial to prevent adverse body composition change in breast cancer patients on adjuvant chemotherapy. Clin Breast Cancer. 2008;8(1):70-9.

9. Haines TP, Sinnamon P, Wetzig NG, Lehman M, Walpole E, Pratt T, et al. Multimodal exercise improves quality of life of women being treated for breast cancer, but at what cost? Randomized trial with economic evaluation. Breast Cancer Res Treat. 2010;124(1):163-75. doi: 10.1007/s10549-010-1126-2.

10. Hammer MJ, Eckardt P, Cartwright F, Miaskowski C. Prescribed Walking for Glycemic Control and Symptom Management in Patients Without Diabetes Undergoing Chemotherapy. Nurs Res. 2021;70(1):6-14. doi: 10.1097/nnr.0000000000000468.

11. He JH, Yao L, Chang Z, Liu GN. Rehabilitation Effect of Systematic Exercise in Adjuvant Chemotherapy f or Breast Cancer. Chinese journal of rehabilitation (zhongguo kang fu). 2011;26(3):204‐6.

12. He X, Ng MSN, Choi KC, So WKW. Effects of a 16-week dance intervention on the symptom cluster of fatigue-sleep disturbance-depression and quality of life among patients with breast cancer undergoing adjuvant chemotherapy: A randomized controlled trial. International journal of nursing studies. 2022;133:104317. doi: <https://dx.doi.org/10.1016/j.ijnurstu.2022.104317>.

13. Hiensch AE, Mijwel S, Bargiela D, Wengström Y, May AM, Rundqvist H. Inflammation Mediates Exercise Effects on Fatigue in Patients with Breast Cancer. Med Sci Sports Exerc. 2021;53(3):496-504. doi: 10.1249/mss.0000000000002490.

14. Huang HP, Wen FH, Yang TY, Lin YC, Tsai JC, Shun SC, et al. The effect of a 12-week home-based walking program on reducing fatigue in women with breast cancer undergoing chemotherapy: A randomized controlled study. Int J Nurs Stud. 2019;99:103376. doi: 10.1016/j.ijnurstu.2019.06.007.

15. Husebø AM, Dyrstad SM, Mjaaland I, Søreide JA, Bru E. Effects of scheduled exercise on cancer-related fatigue in women with early breast cancer. ScientificWorldJournal. 2014;2014:271828. doi: 10.1155/2014/271828.

16. Mijwel S, Jervaeus A, Bolam KA, Norrbom J, Bergh J, Rundqvist H, et al. High-intensity exercise during chemotherapy induces beneficial effects 12 months into breast cancer survivorship. J Cancer Surviv. 2019;13(2):244-56. doi: 10.1007/s11764-019-00747-z.

17. Mock V, Frangakis C, Davidson NE, Ropka ME, Pickett M, Poniatowski B, et al. Exercise manages fatigue during breast cancer treatment: a randomized controlled trial. Psychooncology. 2005;14(6):464-77. doi: 10.1002/pon.863.

18. Mock V, Pickett M, Ropka ME, Muscari Lin E, Stewart KJ, Rhodes VA, et al. Fatigue and quality of life outcomes of exercise during cancer treatment. Cancer Pract. 2001;9(3):119-27. doi: 10.1046/j.1523-5394.2001.009003119.x.

19. Moros MT, Ruidiaz M, Caballero A, Serrano E, Martínez V, Tres A. [Effects of an exercise training program on the quality of life of women with breast cancer on chemotherapy]. Rev Med Chil. 2010;138(6):715-22. doi: 10.4067/s0034-98872010000600008.

20. Mutrie N, Campbell A, Barry S, Hefferon K, McConnachie A, Ritchie D, et al. Five-year follow-up of participants in a randomised controlled trial showing benefits from exercise for breast cancer survivors during adjuvant treatment. Are there lasting effects? J Cancer Surviv. 2012;6(4):420-30. doi: 10.1007/s11764-012-0233-y.

21. Naraphong W, Lane A, Schafer J, Whitmer K, Wilson BRA. Exercise intervention for fatigue-related symptoms in Thai women with breast cancer: A pilot study. Nursing & Health Sciences. 2015;17(1):33-41. doi: 10.1111/nhs.12124.

22. Prakash K, Saini S, Pugazhendi S. Effectiveness of yoga on quality of life of breast cancer patients undergoing chemotherapy: a randomized clinical controlled study. Indian journal of palliative care. 2020;26(3):323‐31. doi: 10.4103/IJPC.IJPC_192_19.

23. Schmidt ME, Wiskemann J, Armbrust P, Schneeweiss A, Ulrich CM, Steindorf K. Effects of resistance exercise on fatigue and quality of life in breast cancer patients undergoing adjuvant chemotherapy: A randomized controlled trial. Int J Cancer. 2015;137(2):471-80. doi: 10.1002/ijc.29383.

24. Schmidt T, Weisser B, Dürkop J, Jonat W, Van Mackelenbergh M, Röcken C, et al. Comparing Endurance and Resistance Training with Standard Care during Chemotherapy for Patients with Primary Breast Cancer. Anticancer Res. 2015;35(10):5623-9.

25. Segal R, Evans W, Johnson D, Smith J, Colletta S, Gayton J, et al. Structured exercise improves physical functioning in women with stages I and II breast cancer: results of a randomized controlled trial. Journal of clinical oncology : official journal of the American Society of Clinical Oncology. 2001;19(3):657-65.

26. Smith-Turchyn J, Richardson J, Tozer R, McNeely M, Thabane L. Bridging the gap: incorporating exercise evidence into clinical practice in breast cancer care. Support Care Cancer. 2020;28(2):897-905. doi: 10.1007/s00520-019-04897-9.

27. Taso CJ, Lin HS, Lin WL, Chen SM, Huang WT, Chen SW. The effect of yoga exercise on improving depression, anxiety, and fatigue in women with breast cancer: a randomized controlled trial. J Nurs Res. 2014;22(3):155-64. doi: 10.1097/jnr.0000000000000044.

28. van Waart H, Stuiver MM, van Harten WH, Geleijn E, Kieffer JM, Buffart LM, et al. Effect of Low-Intensity Physical Activity and Moderate- to High-Intensity Physical Exercise During Adjuvant Chemotherapy on Physical Fitness, Fatigue, and Chemotherapy Completion Rates: Results of the PACES Randomized Clinical Trial. J Clin Oncol. 2015;33(17):1918-27. doi: 10.1200/jco.2014.59.1081.

29. Wang G, Wang S, Jiang P, Zeng C. [Effect of Yoga on cancer related fatigue in breast cancer patients with chemotherapy]. Zhong Nan Da Xue Xue Bao Yi Xue Ban. 2014;39(10):1077-82. doi: 10.11817/j.issn.1672-7347.2014.10.016.

30. Wang Y-J. Effects of a six-week home-based walking program on Taiwanese women newly diagnosed with early stage breast cancer. State University of New York at Buffalo; 2010. p. 142 p- p.

31. Wei X, Yuan R, Yang J, Zheng W, Jin Y, Wang M, et al. Effects of Baduanjin exercise on cognitive function and cancer-related symptoms in women with breast cancer receiving chemotherapy: a randomized controlled trial. Supportive care in cancer. 2022;30(7):6079‐91. doi: 10.1007/s00520-022-07015-4.

32. Yang CY, Tsai JC, Huang YC, Lin CC. Effects of a home-based walking program on perceived symptom and mood status in postoperative breast cancer women receiving adjuvant chemotherapy. J Adv Nurs. 2011;67(1):158-68. doi: 10.1111/j.1365-2648.2010.05492.x.
